# Identifying features of source and message that influence the retweeting of health information on social media during the COVID-19 pandemic

**DOI:** 10.1186/s12889-022-13213-w

**Published:** 2022-04-22

**Authors:** Jingzhong Xie, Liqun Liu

**Affiliations:** 1 School of Journalism and Communication, Wuhan University, Wuhan, 430072 China; 2 National Institute of Cultural Development, Wuhan, University, Wuhan, 430072 China; 3 Center for Studies of Media Development, Wuhan University, Wuhan, 430072 China

**Keywords:** China, COVID-19, Health Information, Infodemiology, Information Dissemination, Information Frame, Retweeting, Social Media, Weibo, Zero-Inflated Negative Binomial

## Abstract

**Background:**

Social media has become an essential tool to implement risk communication, giving health information could gain more exposure by retweeting during the COVID-19 pandemic.

**Methods:**

Content analysis was conducted to scrutinize the official (national and provincial) public health agencies’ Weibo posts (*n* = 4396) to identify features of information sources and message features (structure, style content). The Zero-Inflated Negative Binomial (ZINB) model was adopted to analyze the association between these features and the frequency of the retweeted messages.

**Results:**

Results indicated that features of source and health information, such as structure, style, and content, were correlated to retweeting. The results of IRR further suggested that compared to provincial accounts, messages from national health authorities’ accounts gained more retweeting. Regarding the information features, messages with hashtags#, picture, video have been retweeted more often than messages without any of these features respectively, while messages with hyperlinks received fewer retweets than messages without hyperlinks. In terms of the information structure, messages with the sentiment (!) have been retweeted more frequently than messages without sentiment. Concerning content, messages containing severity, reassurance, efficacy, and action frame have been retweeted with higher frequency, while messages with uncertainty frames have been retweeted less often.

**Conclusions:**

Health organizations and medical professionals should pay close attention to the features of health information sources, structures, style, and content to satisfy the public’s information needs and preferences to promote the public's health engagement. Designing suitable information systems and promoting health communication strategies during different pandemic stages may improve public awareness of the COVID-19, alleviate negative emotions, and promote preventive measures to curb the spread of the virus.

## Background

Breaking out at the end of 2019, COVID-19 was first identified in Wuhan, China, and then been reported worldwide. World Health Organization (WHO) declared the outbreak of COVID-19 a pandemic on 11th March 2020. Apart from effective medical research and practice, competent communication strategies regarding such infectious diseases have played a pivotal role in preventing global outbreaks when dealing with a pandemic [1]. Since communication can construct people’s consciousness and impact ones’ judgment on the current situation [2]. However, false and misleading information in media, especially social media, may also lead to a negative perception of public health risks [3]. The general public is more likely to take practical measures to protect health through transparent and up-to-date communication strategies [4].

China, the first nation defending itself against COVID-19, has taken a series of actions, including reporting on and informing the pandemic situation consistently, to prevent the virus from spreading all over the country. Apart from traditional media, such as radio, television, and print channels, public health institutions also used a variety of social media, including government micro-blogs. By providing authentic and updated information on these social media, public health authorities aimed to eliminate fake information, alert the general public and advocate proper prevention measures. In particular, under the timing of national-wide quarantine, personal and group communication was limited. Compared with any previous health events, these information services of health works played a more significant part during COVID-19.

Nowadays, social media, where users seek and exchange health information, have been increasingly popular and provided a tremendous amount of information, including health-related information. Under a health crisis, social media has become the primary channel for health communication. They have been globally adopted by the Centers for Disease Control and Prevention (CDC) and related government agencies to inform the public about new communicable diseases [5, 6]. Unlike traditional media, social media enables health organizations to deliver health information to large audience in a shorter period by retweeting and sharing the information [7]. Social media also extend the boundary of health-related information by prompting content sharing behaviors among users [8].

Although increasing numbers of public health organizations are convinced by the value of social media, such as Twitter, Facebook, and Weibo, for health communication [9]. They have not yet achieved the full potential of these platforms [10]. The major contributing factor resides in that a large number of public health organizations have been using social media as a one-way information channel rather than taking their users’ needs and preferences into consideration [11]. Therefore, the exposure of health information shared by these organizations is often insufficient. The ultimate purpose of health communication is persuasion, whose precursor is exposure [12], a predictor that determines whether information alters the audience’s behaviors. It is critical to identify the factors that influence the retweeting of health information, better understanding users’ needs and preferences of health information shared in social media, during emergencies, is of practical importance.

Similar to Twitter, Sina Weibo is the most influential and popular social media platform in China. According to its financial report in the third quarter of 2020, it has 511 million monthly active users and 224 million average daily active users in September 2020 [13]. In recent studies about COVID-19, Chen et al. investigated Healthy China’s Weibo posts and then suggested that the message with high media richness and positive emotion is likely to increase citizen’s engagement through government social media [14]. Ngai et al. analyzed COVID-19 related messages posted in People's Daily Weibo and found that the content, message style, and interactive features affect the public’s engagement [15]. However, these studies only focused on national-level organizations or specific accounts. Liao argued that at the early stage of COVID-19, the most active government agencies’ Weibo accounts were province-level, and even at the city level accounts [16]. In this study, we developed a conceptual model considering the features of source and message, to empirically examine the driving factors that impact the public's retweeting of Weibo messages from Chinese public health agencies, during the initial phase of the COVID-19 pandemic.

### Literature review

Retweeting, an important information dissemination mechanism has drawn attention from a broader range of scholars to discuss the factors that encourage retweeting behaviors [17–20]. These studies have shown that the influences created by sources and content features are always significant. Thus, this study focus on discussing the impacts of source features and message features (structure, style, and content).

### The feature of source

A message is initially sent by some individual senders and those senders’ characteristics still are fundamental to the process of message diffusion [21]. Previous studies of retweeting behaviors of Twitter users have indicated that user identity and the number of followers are critical factors to determine whether a tweet will be retweeted [22]. In addition, other researchers have found sender-level characteristics also influence the passing of information [20, 23]. For example, during the Ebola crisis, Vos et al. found that Twitter messages shared by state and federal public health organizations are retweeted more than that of local counterparts. In China, the public health agencies’ credibility is positively correlated with the level of organizations. In other words, the higher-level official organization generally possessed a stronger sense of authority [24]. Hence, based on the features of sources and mechanism of retweeting function, we raised the following research question:

- **RQ1: What source features of health information in Weibo, (a) the number of account followers, (b) the type of account, if any, will affect message re-tweeting?**

### Message features

Previous empirical investigations have supported the positive correlation between message passing and message features [19, 25], which were classified as the structural, stylistic, and content factors [26]. Naveed et al. attempted to categorize these factors into low level and high-level [18]. When it comes to the low-level ones, they usually are directly extracted from the message without further processing and contain URLs, hashtags, usernames, and question and exclamation marks. In terms of high-level ones, they are often associated with the topic, the sentiments, and the stances of the tweets.

#### Structure features

URL, hashtag, and the mention of username are the micro-structural elements [19] and particular functions of Twitter and Weibo [18]. To be more specific, URL allows tweets beyond the limitation of 280 characters and connects to other websites for further information. Therefore, health agencies can make full use of URLs to offer credible and detailed information [27]. Additionally, hashtag#, words or phrases without spaces, enables users to find relevant tweets or a topic and engage in newly emerging social media conversation. In addition, the function of @username is an attention-seeking method and an opportunity to notify the person being talked about.

Compared with text-only tweets, tweets containing graphics can draw users’ attention, according to McCaffery et al. in 2012 [28]. Relevant research has proven that pictures have significantly encouraged more retweeting and discussing health messages on Twitter [23] and Facebook [17]. Therefore, we raised research question:

- **RQ2: What structure features of health information in Weibo, (a) hyperlink, (b) #hashstag, (c) @mention of username, (d) pictures, and (f) video, if any, will affect message retweeting?**

#### Stylistic features

Apart from those elements, the variation in rhetoric, personal pronouns, and linguistic characteristics will impact the delivery of the information and audiences’ mental reaction. The presence of punctuation marks, for example, is a good indicator of senders’ emotional judgment but is often neglected [29]. A prior study in 2011 has statistically demonstrated that exclamation marks convey strong emotional statements, and question marks express questioning emotion naturally intended to elicit responses [18]. Further research also agreed with previous studies that emotional content is more likely to be viral on social media [30]. Thus, we ask:

- **RQ3: What stylistic features of health information in Weibo, (a) Q&A and (b) emotional, if any, will affect message re-tweeting?**

#### Content features

In 1993, Entman came up with the frame theory that to ‘frame’ is to emphasize certain aspects of a communicating text [31], and Gamson et al. suggested that the frame is the centrality of news stories [32]. For example, journalists might use the frame to maximize or hide certain opinions or messages to define current issues in reporting health-related news. However, experts are concerned that non-experts are likely to be influenced and then underestimate health risks. In 2018, Dan and Raupp [33] did a systematic review on 37 articles on health-risk news reporting and summarized 15 different frames, including a health risk frame, responsibility frame, action frame, and so on, which are widely used in reporting on pandemics, such as flu, SARS, and AIDS.

We have discussed the low-level features of social media (Twitter and Weibo) above, but high-level features, usually referring to the topic, sentiments, and stances, also have an indispensable effect in information delivery processes. Previous research has supported the correlation between the high-level features and information dissemination during healthy emergent crises, such as the 'health belief model' (HBM) [34], 'threat', and 'efficacy' [23]. In this study, frame theory will be utilized to analyze public health agencies’ Weibo posts. Drawing on Entman’s three basic functions of the frame, we selected 6 frames out of the 15 frames to achieve these functions of the frame, including defining problems, identifying their respective causes, and proposing solutions/moral evaluation, to identify which information content with the different framework is more likely to be retweeted. Thus, we propose the following research question:

- **RQ4: What content features of health information in Weibo, (a) severity framework, (b) action framework, (c) efficacy framework, (d) uncertainty framework, (e) reassurance framework, and (f) technology framework, if any will affect message retweeting?**

## Methods

### Sampling and data collection

An automated web scraping tool was built in the programming language Python to collect public posts on Sina Weibo in the Chinese language from the 23^rd^ January to the 22^nd^ February 2020. Data were collected from 41 authorized Sina Weibo accounts which contained most provincial public Health Commission (HC) and CDC of mainland China. The reasons why this period time was chosen lie in that (1) on January 23rd, the Wuhan citywide quarantine had become a significant public opinion event, causing a nationwide concern, followed by a series of first-level responses to pandemic situations initiated by every province; (2) on February 22nd, President Xi made a speech, indicating that China had entered a stage where pandemic prevention and control should align with work resumption. Therefore, during this period time, the frequency of sharing messages about pandemic prevention and control was high.

We set the Covid-19 related keyword to collect data continuously, included [新型冠状病毒] (novel coronavirus), [武汉肺炎] (Wuhan pneumonia), [不明原因肺炎] (un-known caused pneumonia), [疫情] (epidemic situation), [非典] (severe acute respiratory syndrome), [华南海鲜市场] (Wuhan Seafood Wholesale Market). Data collected included textual content of the post, account information, date and time of post, and tagged with pictures and videos. There were 33 Weibo accounts, among 41 official government accounts, which posted related messages, totaling 4396 messages ( see Table 1).

**Table 1:** Provincial and National Public Health Agencies Included in the Data Set

Agency Name	No. of Messages	Agency Name	No. of Messages
National HC	509	Guizhou HC	125
National CDC	139	Guizhou CDC	87
Hebei HC	154	Yunnan HC	138
Hebei CDC	50	Yunnan CDC	84
Liaoning HC	97	Shannxi HC	55
Jilin HC	122	Gansu HC	218
Jiangsu HC	233	Guangxi HC	509
Anhui HC	84	Guangxi CDC	18
Fujian HC	169	Tibet HC	7
Jiangxi HC	56	Ningxia HC	4
Shandong HC	335	Xinjiang HC	1
Henan HC	117	Beijing HC	101
Henan CDC	3	Beijing CDC	60
Hunan CDC	32	Tianjing HC	269
Guangdong HC	231	Shanghai HC	147
Sichuang HC	229	Chongqing HC	7
Sichuang CDC	6		

### Operationalization of variables

#### Retweeting

As the outcome variable, retweeting was measured by the frequency of messages from Chinese public health agencies.

#### Source features

Source features include the number of account followers and account types. The number of followers is counted by the actual number of followers of each source account. Account types are divided into two types: national health organizations and provincial organizations, and are coded as 1 and 2 respectively.

#### Structure features

Five items are used to measure structure features, including the use of hyperlinks, hashtag#, mention of username @, photos, and videos. Each of the items is coded as either 1 or 0, with 1 being a positive response.

#### Stylistic features

Stylistic features measured by the use of question mark (?) and exclamation mark (!). Similarly, each of the items is coded as either 1 or 0.

#### Content features

Based on the study of Dan and Raupp [33], this study classifies the content features into six frames, namely severity, reassurance, efficacy, uncertainty, action, and technology. Each of the items is coded as either 1 or 0.

### Coding procedure and reliability

Based on the operationalization of variables above, the messages were coded from the source, structure, style, and content and each Weibo post was the units for coding. The codebook has been shown in Table2. Although codes were distinctive, they were not mutually exclusive. Therefore, one message is likely to have multiple features. In addition, the relevant codes to the programming language were added in Python 3.8.1 to determine whether it contains source features, structural features, and style features. At the same time, the content features were coded manually.

**Table 2:** Coding strategy, Variables Definitions, Intercoder Reliability, Examples

	**Variables**	**Definition**	**Intercoder Reliability**	**Application examples**
Structure	Hyperlink	With a hyperlink to another website	-	National Health Commission’s announcement to satisfy the basic health demand of the general public, link:http://www.nhc.gov.cn/yzygj/s7659/202002/6d5a8556c5ce46368263711698d8237a.shtml
	#	with the hashtag #	-	Guidance for New Coronavirus Prevention #New Coronavirus Prevention##New Coronavirus Pneumonia Prevention#
	@	Mention an another individual or organization’s name	-	#New Coronavirus# What can you do if you got pneumonia? Protect yourself and protect others @Health China @People’s Daily @National Health 12,320 @ Weibo Health
	Picture	With a picture	-	#Pop Health Science# Guidance for Pregnant Women and Children to prevent Corona Virus (cartoon version) [picture]
	Video	With a video	-	#Pop Health Science# During the time period of pandemic, how should parents help children to physiologically adjust? [video]
Style	Q&A (?)	With question mark? to indicate a question and an answer	-	Am I a close contact? Don’t “underestimate” the enemy! Whom should be counted as a close contact!
	Emotional (!)	With exclamation mark! to indicate a strong sentiment	-	Attention! In the face of pandemic, we can’t ignore mental health!
Content	Severity frame	With the information of the severity of the disease, including the incidence of disease, and mortality etc	0.89	#Nationwide Confirmation of Coronavirus Cases# Till 22^nd^ January, NHC has reported with 571 confirmed cases, 95 serve cases, 17 death cases (all from Hubei), and 393 suspected cases
	Uncertainty frame	With the information of the uncertainty of the disease, including the defining the unknown mechanism of the virus and the different opinions among experts	0.86	#cured patients returned positive after nucleic acid amplification testing# Experts are still figuring out why cured patients return positive result
	Action frame	To announce governmental action to combat the disease, including important meeting, policy, inspection, and punishing officials who neglected their duties to prevent and control the disease	0.84	#Health Announcement# The CCP central committee has set up a specific group to fight against the disease
	Reassurance frame	To encourage the public not worry and increase the confidence over governments’ solutions, including the preparation, medical experts’ contribution, the stories of gross-root units, the number of cures, and donation or encouragement from individuals and organizations	0.87	#Gross-roots Fight against the Disease# A group of voluntary medical workers is helping firms and corporations with reopening
	Efficacy frame	To emphasize the specific solutions for individuals and organizations, including the interpretation of experts and guidance	0.87	#Pop Health Science# China Central Television’s anchors teach you how to select the proper facial masks
	Technology frame	Known medical information and treatment breakthrough	0.82	#New Coronavirus # What is fecal–oral transmission?

*Note*. Reliability is measured using Krippendorff’s alpha (α)

To evaluate the reliability of manual coding, two well-trained coders randomly selected 15% Weibo posts (*n* = 660) to run the coding test and the reliability was assessed with Krippendorff’s alpha, which was calculated by the KALPHA macro for SPSS [35]. As a result, the intercoder reliability was between 0.82 and 0.89, suggesting strong reliability.

### Statistical analysis

The outcome variable in this study was set as the frequency of messages from Chinese public health agencies being retweeted and containing non-negative integer values. For such variables, the most common way is to build a Poisson regression model. However, the Poisson regression model requires that the mean equals the variance. Unfortunately, the discreet (count) data for the outcome variable in this study was over-dispersed, the concentration of the values around zero was disproportionately higher, and the variance was significantly larger than the mean (M = 45.67, SD = 281,264). Due to these conditions, we used Zero-Inflated Negative Binomial (ZINB) regression models to examine which factors predicted the frequency of retweeted messages, particularly as it presents overdispersions and can adequately handle issues related to the presence of many zero values [36]. In addition to testing the significance of coded variables, including structural features, style features, and content features, we also examined the association between the frequency of retweeted messages and features of information sources. All independent variables are classified variables, except the number of followers. Data analysis was conducted using Stata 15.1 Program.

## Results

### Descriptive analysis

As shown in Table 3, we statistically analyzed 4396 messages. Among these messages, 76.3% were retweeted at least once. The maximum and the minimum number of followers of microblog accounts were 5,615,973 and 360. In this study, 85.3% of the messages were released by provincial health institutions, and the rest came from national-level health institutions. 48.1% of the messages were with “#COVID-19”, and 37.2% contained photos. The reassurance frame (37.7%), efficacy frame (29.0%), and severity frame (26.8%) were mainly used by Chines official medical institutions for information dissemination.

**Table 3:** Descriptive statistics for study variables (*N* = 4396)

Variable	N (%)	Mean (SD)	Min	Max
Retweet	3355 (76.3%)	45.67(281,264)	0	32,744
Followers		566,393(1.732E + 12)	360	5,615,973
Governmental level				
National	648 (14.7%)			
Provincial	3748 (85.3%)			
Hyperlink	1647 (37.5%)			
#	2116 (48.1%)			
@	329 (7.5%)			
Picture	1635 (37.2%)			
Video	429 (9.8%)			
Q&A (?)	683 (15.5%)			
Emotional (!)	1139 (25.9%)			
Frame				
Severity	1179 (26.8%)			
Uncertainty	12 (0.3%)			
Action	608 (13.8%)			
Reassurance	1656 (37.7%)			
Efficacy	1275 (29.0%)			
Technology	141 (3.2%)			

### Multicollinearity test

In order to rule out a linear relationship among genres of information and improve the fitting accuracy, we ran the multicollinearity test before building the model. It is widely known that the stronger the collinearity among explanatory variables, the larger the variance inflation factor (VIF) is. The result of the test has shown that the mean of major explanatory variables is acceptable, indicating that the collinearity is not significant.

### ZINB model results

Table 4 chiefly presented the correlation coefficient among explanatory variables. From it we can see that the absolute value of the correlation coefficient among explanatory variables is less than 0.5, supporting the independence in different variables, which could be used in fitting models.

**Table 4:** Correlation Coefficiency among Key Explanatory Variables

	Severity	Reassurance	Efficacy	Uncertainty	Action	Technology
Severity	1					
Reassurance	-0.318	1				
Efficacy	-0.167	-0.457	1			
Uncertainty	-0.012	-0.023	-0.024	1		
Action	-0.232	-0.135	-0.229	-0.008	1	
Technology	-0.093	-0.128	-0.040	0.238	-0.062	1

To judge whether there is Zero-Inflation, we need to analyze the proportion of zeros first. After data analysis, the proportion of zeros in “uncertainty”, “action”, and “technology” is the highest, surpassing 70%. The feature of zero-inflation surfaced and thereby we decided to use zero-inflated Poisson regression to run the empirical test.

Table 5 showed the estimated results calculated by the ZINB model. To control the in-fluence brought by different periods, the model of this study controlled the everyday fixed time effect. The estimated results showed that followers (Β = 0.297, *p* = 0.000), governmental level (Β = 2.055, *p* = 0.000), hashtags# (Β = 0.225, *p* = 0.007), picture (Β = 0.476, p = 0.000), video (Β = 0.588, *p* = 0.000), emotional(!) (Β = 0.419, *p* = 0.000), severity (Β = 1.983, *p* = 0.000), reassurance (Β = 0.532, *p* = 0.000), efficacy (Β = 0.609, *p* = 0.000) and action (Β = 14.274, *p* = 0.000) were associated with increased message retweeting, while hyperlink (Β = -1.272, *p* = 0.000) and uncertainty (Β = -16.193, *p* = 0.000) correlated with reduced message retweeting. In Addition, @ (Β = -0.099, *p* = 0.413), Q&A(?) (Β = -0.153, *p* = 0.155) and technology (Β = 2.767, *p* = 0.104) had no association with message retweeting.

**Table 5:** The Estimated Result of ZINB model

Predictor	B	S.E.	*P*	IRR [95% CI]	Sig.
**Source Feature**					
Followers(log)	0.297	0.019	0	1.345 [1.295 1.398]	***
Governmental level	2.055	0.121	0	7.809 [6.161 9.897]	***
**Message Feature**					
Structure					
Hyperlink	-1.272	0.077	0	0.280 [0.241 0.326]	***
@	0.099	0.121	0.413	1.104 [0.871 1.399]	NS
#	0.225	0.083	0.007	1.252 [1.0645 1.475]	**
Picture	0.476	0.078	0	1.610 [1.382 1.875]	***
Video	0.588	0.146	0	1.800 [1.352 2.397]	***
Style					
Q&A(?)	-0.153	0.108	0.155	0.858 [0.694 1.060]	NS
Emotional(!)	0.419	0.088	0	1.521 [1.279 1.808]	***
Content					
Severity	1.983	0.095	0	7.267 [6.029 8.759]	***
Reassurance	0.532	0.106	0	1.703 [1.383 2.096]	***
Efficacy	0.609	0.01	0	1.839 [1.513 2.235]	***
Uncertainty	-16.193	1.962	0	-16.194 [-20.038 -12.349]	***
Action	14.274	0.827	0	14.274 [12.654 15.894]	***
Technology	2.767	1.702	0.104	2.7668 [-0.5683 6.1018]	NS

*Note*: *NS* Not significant; * *p* < 0.05; **p < 0.01; ***p < 0.001

To explain our findings, we have provided a frequency interpretation of the results based on the incident rate ratios (IRR) [36]. Frequency effects are additive on a logarithmic scale, suggesting that if one message possesses multiple features, each feature is more likely to lead to more message sharing.

In the feature of sources, the IRR result showed that with other variables remaining unchanged, a one-point increase in the percentage of followers was associated with a 34.5% increase in message retweeting (IRR = 1.345; 95% CI 1.295—1.398). Messages from the national agencies were retweeted 680.9% more often than provincial counterparts (IRR = 7.809; 95% CI 6.161—9.897).

The features of information structure, hyperlink, hashtags#, images, and videos were all significantly were associated with the retweeting. Specifically, with other variables remaining the same, messages that included hashtags#, pictures and video were retweeted 25.2% (IRR = 1.252; 95% CI 1.064—1.475), 61.0% (IRR = 1.610; 95% CI 1.382—1.875), and 80.0% (IRR = 1.800; 95% CI 1.352—2.397) more often than messages without these features respectively. However, messages that included hyperlinks were retweeted 72% (IRR = 0.280; 95% CI 0.241—0.326) less often than messages without hyperlinks. Furthermore, @ did not significantly impact retweeting.

In the features of message style, messages with a sentiment (!) were retweeted 52.1% (IRR = 1.521; 95% CI 1.279—1.808) more often than messages without sentiment, while no significant effect in question (?) was found.

With six variables of information content, messages containing severity frame, reassurance frame, efficacy frame and action frame were retweeted 626.7% (IRR = 7.267; 95% CI 6.029—8.759), 70.3% (IRR = 1.703; 95% CI 1.383—2.096), 83.9% (IRR = 1.839; 95% CI 1.513—2.235), and 1327.4% (IRR = 14.274; 95% CI 12.654—15.894) more often than messages without these frames. Uncertainty, however, was significant and negative. Messages containing uncertainty frames were retweeted 1719.4% (IRR = -16.194; 95% CI -20.038—-12.349) less often than messages without uncertainty frames. Technology was not significantly associated with retweeting.

## Discussion

This study attempted to identify the factors that influenced the retweeting of health information on social media during the COVID-19 pandemic in China. To reveal the mechanisms of retweeting, we built on the prior studies to modify and test a communication model, which investigated the association between source features, message features (including structure features, style features, and content features), and retweeting of health messages.

In terms of RQ1, the study has shown that messages posted on higher-level Weibo accounts with more followers are easier to forward. The result supported previous findings which claimed that accounts with many followers have the highest spread [37]. This shed light on the importance of national public health agencies in health communication. This result might be explained by the fact the credibility of the message is another significant variable in health communication, particularly under the circumstance of the increasing amount of unreliable information shared on social media platforms [38]. In the case of health communication, authoritative sources, such as doctors, can be more credible and more persuasive than non-expert sources [39, 40]. Therefore, more followers and a higher level mean more professional knowledge and credibility. During a time of high uncertainty in a pandemic, national health agencies with more followers are the most trusted sources of COVID-19 related information [41].

To RQ2, it was found that hyperlinks and hashtags# were associated with Weibo retweeting. Specifically, hashtags# related to novel coronavirus promoted the retweeting, consistent with previous studies about Zika [20] and Ebola [23], especially a recent study examining Canadian COVID-19 tweets [42]. A possible explanation might be that during public health crises, the function of hashtags# enables users to locate the relevant information and increase the interaction. On the contrary, hyperlinks tended to reduce the Weibo retweeting, agreeing with Sutton’s finding [19]. Although Vos et al. pointed out that in the Ebola and Zika pandemics study, hyperlinks would not increase or decrease the information retweeting [20]. Therefore, using a hyperlink is a more complicated information strategy that the motives and circumstances of using hyperlinks should be taken into further consideration.

Additionally, consistent with prior studies about Facebook, this research found that more visual cues such as photos or videos could increase the information retweeting generate more engagement [43], probably not only because photos and videos are easier to understand than plain text [44], but also because they signal greater credibility of information [45]. The plain and straightforward language shared by social media makes information easily accessible to laypeople [46]. Pictures and videos with epidemic-related information are the first choices for the public who do not have health professional knowledge. In general, pictures and videos could make it more direct and clear to understand what COVID-19 is and how to do preventive measures.

Another finding was that information style is an important design strategy determining Weibo retweeting. Namely, information with a robust emotional style is more likely to be retweeted. This result reflected some previous studies found that information with different emotions, such as humor or disgust, tends to increase retweeting [8, 47]. Therefore, information style, a rhetorical strategy, should be taken into consideration for social media professionals. Meanwhile, according to an investigation of Healthy China's Weibo posts, the message with both high media richness and positive emotion is likely to increase citizen engagement through government social media during the COVID-19 pandemic [14]. Therefore, as a rhetorical strategy, information style should be valued by health information professionals to cope with the different stages of the pandemic. In the early stage of the pandemic, Q&A style information may be urgently needed by the public, while in the progress stage of the pandemic, emotional style information can mobilize public participation and enhance confidence in prevention and control.

It was interesting to note that by examining the Weibo content posted by general CDCs and HCs, organizations tend to use reassurance and efficacy frames to disseminate information about “typical success stories of doctors on the ground” and “effective achievements of anti-COVID-19 efforts”. This empathic style of communication could grab public attention, calm down the public and address health concerns [48]. Conversely, the uncertainty frame was seldom utilized in these messages, possibly because they do not wish to cause unnecessary panic in the community. Another critical finding resided in the different frames utilized between nation-level and province-level agencies. Specifically, 30.5% (*n* = 1145) of Weibo content used risk frames by province-level agencies. By contrast, only 5.2% (*n* = 34) used by national-level agencies, indicating that local agencies were more concerned about the risks to the local public health. Additionally, compared with province-level agencies, national-level ones preferred action frames, with 23% (*n* = 149) and 12.2% (*n* = 459) respectively, because national agencies were more willing to magnify the importance of governments.

Unlike earlier studies, this study started with the information frame, examined the information content presented by Chinese public health agencies, and explored the contributing factors of Weibo’s retweeting (RQ4). The study found that severity, assurance, medical (efficacy information), and action frames increase the retweeting of information. This result aligned with the prior studies that information containing governmental actions, medical messages, severity, and efficacy had higher chances of being retweeted [20]. News stories with assurance information could reach broader audiences than stories with warning information in emerging pandemics [49].

The study also suggested that a few usages of uncertainty frame did not increase the retweeting behaviors. However, the uncertainty frame and reassurance frame provided the ground to make a moral evaluation about the risk, because they give the general public clues to judge whether the future is secured or uncertain [33]. As a new type of coronavirus, there existed uncertainties in the early stage of COVID-19, not only in the medical field, but the general public has very little awareness of it. Therefore, in the early days of the spread of the COVID-19, the public’s fear and anxiety for the unknown nature of the coronavirus is dominant on social media [50]. The reason why the uncertainty frame discourages Chinese social media users from retweeting probably could be explained by the uncertainty avoidance from Hofstede's Culture Dimensions Theory [51], referring to how comfortable a person feels under an unstructured environment. Shanks et al. confirmed that uncertainty avoidance is a major component of Chinese society [52], and Nitish et al. also found that the frequency of uncertainty avoidance in Chinese websites was higher than that of American domestic websites [53]. Therefore, it is wise for public health agencies not to use such a strategy in reporting pandemics, especially in the early stage of COVID-19.

Noteworthy, the results of IRR showed that, in general, the message content was the most prominent feature affecting message retweeting, followed by source, structure, and style features. Namely, the content of the tweet (e.g., the frame) is more likely to generate more sharing and retweeting than the source (e.g., the number of followers) or platform features (e.g., hashtags #, mention of username@). This indicated that the information function was the most prominent function among the studied tweets [54]. Tweets with informational features were more likely to be retweeted in the early stages of an infectious disease outbreak characterized by a lack of information and a high degree of uncertainty.

## Conclusions

More exposure through retweeting on social media provides public health departments to deliver health information to a larger audience as soon as possible, which establishes the interaction and communication with the public. However, factors affecting the retweeting of health information are not completely apparent. This study provided an insight into this field by the analysis of 4396 messages posted by public health agencies in China and discusses what impacts the retweeting of health messages. The study showed that retweeting was significantly correlated to the source features and information features.

These findings make several contributions to the current research and reality. At first, the active use of social media has demonstrated the value of Infodemiology and Infoveillance during the early stage of the outbreak of COVID-19 [55]. To get rid of the top-down and one-way approach in risk communication, summarized by Liao in 2020 [9], government agencies should obtain instant feedback from the ground up by tracking the direct responses online and then taking timely measures. Secondly, the structure, style, and content are key components to determine the information retweeting on social media (Weibo). Therefore, health communication professionals should share the customized message on social media to satisfy the information needs and preferences of the audience in different stages of the pandemic. Particularly, in the current immunization campaigns, the government agencies not only need to understand the acceptance and information needs of different populations for vaccines to formulate effective communication strategies to prevent misinformation, but also consider the audience’s emotional dynamics, including panic, anxiety, and sympathy, to provide emotional supports. Thirdly, the local information sources should be more valuable since the message of province-level agencies is more related to the local audience. The education and training of health library and information workers should be organized and developed rapidly in local community organizations, to better interact with local followers.

### Limitations

However, there are some limitations to this research. Firstly, the messages are shared only by the provincial and national public health agencies, instead of more messages from grassroots agencies and news media, and without considering the existence of social media robots. In addition, the results of the study can only be used as a reference for the staged release of information in social media, as samples only included data from the early stages of the pandemic, and did not cover every phase of COVID-19’s development, especially current virus immunization campaign. Another limitation in the study was that it only examined the re-tweeting of social media messages and did not pay attention to the comments and likes of social media information. They are an important part of public engagement on social media. Finally, whether Weibo’s retweeting mechanism could be used by more private social media platforms (such as Wechat) and different cultural settings (collectivism and individualism) should be further tested.

## Data Availability

The data that support the findings of this study are available from Sina Weibo. Restrictions apply to the availability of these data used under the license for this study. Data are available from the authors with the permission of Sina Weibo.

## Abbreviations

ZINB: Zero-Inflated Negative Binomial
WTO: World Health Organization
CDC: Centers for Disease Control and Prevention
HC: Health Commission
M: Mean
SD: Standard deviation
SE: Standard error
IRR: Incident Rate Ratios
